# LINC01526 Promotes Proliferation and Metastasis of Gastric Cancer by Interacting with TARBP2 to Induce GNG7 mRNA Decay

**DOI:** 10.3390/cancers14194940

**Published:** 2022-10-09

**Authors:** Jin-Yong Zhou, Jin-Yan Liu, Yu Tao, Chen Chen, Shen-Lin Liu

**Affiliations:** 1 Jiangsu Province Hospital of Chinese Medicine, The Affiliated Hospital of Nanjing University of Chinese Medicine, Nanjing 210029, China; 2 Department of Breast and Thyroid Surgery, The Affiliated Suzhou Hospital of Nanjing Medical University, Suzhou Municipal Hospital, Gusu School, Nanjing Medical University, Suzhou 215002, China

**Keywords:** gastric cancer, lncRNA, LINC01526, TARBP2, GNG7, mRNA stability

## Abstract

**Simple Summary:**

Many long noncoding RNAs play an important role in gastric cancer progression. In this study, we focused on LINC01526. Through expression and functional analyses, we obtained a preliminary understanding of the pro-cancer role of LINC01526 in gastric cancer. Furthermore, RNA pull-down and RNA immunoprecipitation chip assays demonstrated that LINC01526 interacts with TARBP2, an RNA-binding protein controlling mRNA stability. Moreover, TARBP2 could bind and destabilize GNG7 transcripts. Finally, the rescue assay disclosed that LINC01526 promoted gastric cancer progression by interacting with TARBP2, leading to the degradation of GNG7 mRNA.

**Abstract:**

Gastric cancer is the most common malignancy of the human digestive system. Long noncoding RNAs (lncRNAs) influence the occurrence and development of gastric cancer in multiple ways. However, the function and mechanism of LINC01526 in gastric cancer remain unknown. Herein, we investigated the function of LINC01526 with respect to the malignant progression of gastric cancer. We found that LINC01526 was upregulated in gastric cancer cells and tissues. The function experiments in vitro and the Xenograft mouse model in vivo proved that LINC01526 could promote gastric cancer cell proliferation and migration. Furthermore, LINC01526 interacted with TAR (HIV-1) RNA-binding protein 2 (TARBP2) and decreased the mRNA stability of G protein gamma 7 (GNG7) through TARBP2. Finally, the rescue assay showed that downregulating GNG7 partially rescued the cell proliferation inhibited by LINC01526 or TARBP2 silencing. In summary, LINC01526 promoted gastric cancer progression by interacting with TARBP2, which subsequently degraded GNG7 mRNA. This study not only explores the role of LINC01526 in gastric cancer, but also provides a laboratory basis for its use as a new biomarker for diagnosis and therapeutic targets.

## 1. Introduction

Over the past decade, gastric cancer (GC) has been a major contributor to global cancer cases. With the reduction in H. pylori infections and the improvement of quality of life, the incidence of GC has gradually decreased [1]. However, the impact of GC on human health should not be underestimated, as GC still ranks fifth (5.6%) in incidence and fourth (7.7%) in mortality globally, according to the GLOBOCAN 2020 Global Cancer Statistics [2]. The occult nature of the early clinical symptoms of GC and the absence of invasive gastroscopy hinder the diagnosis of early GC; moreover, the complexity of the disease and the emergence of drug resistance limit the treatments’ effects [3,4,5,6]. Thus, the search for new GC biomarkers and therapeutic targets is particularly important.

In recent years, long noncoding RNAs (lncRNAs) have gained increasing attention as a cancer biomarker for early screening, diagnosis, prognosis, and the response to drug therapy [7,8,9]. LncRNAs, a heterogeneous set of polyadenylated RNAs longer than 200 nucleotides without a protein-coding capacity [10,11], extensively participate in various physiological and pathological processes [12,13,14,15], including tumorigenesis [16,17,18]. Many lncRNAs play crucial oncogenic or tumor-suppressant roles and regulate the initiation and progression of GC. For instance, GC patients have remarkably elevated levels of LINC01503, which mediates cell cycle progression and tumorigenesis [19]; SNHG22 promotes the proliferation and invasion of GC [20]; patients with chemotherapy-resistant GC express high EIF3J-DT levels [21]; meanwhile, SEMA3B-AS1 is downregulated in gastric cardia adenocarcinoma and suppresses tumor progression [22]. Overall, the role of lncRNAs in the development of GC and its molecular mechanism deserve to be explored.

In a recent study, Cheng et al. have established a prognostic signature for predicting the disease-free survival in GC patients [23]. They suggested LINC01526 as a prognostic marker for GC [23], but the function of LINC01526 has not been explored. The current study was designed to investigate the carcinogenic effect of LINC01526 in GC and the underlying mechanism involved in it. We found that LILNC01526 was highly expressed in gastric cancer tissues and cells, and LINC01526 overexpression was associated with a poor prognosis for GC patients. Moreover, loss-of-function assays indicated that LINC01526 could promote GC cell proliferation and migration in vitro and in vivo. Finally, the exploration of the mechanism revealed that LINC01526 interacts with TAR (HIV-1) RNA-binding protein 2 (TARBP2) and thereby destabilizes G protein gamma 7 (GNG7) mRNA. In summary, this study describes the role of LINC01526 in GC progression and suggests a potential biomarker for the detection or prognosis of GC.

## 2. Materials and Methods

### 2.1. Bioinformatics Analysis

We obtained clinical and genetic expression data from tissues of 32 normal subjects and 375 GC patients from The Cancer Genome Atlas (TCGA) dataset and analyzed them as previously described [24]. Moreover, we used TEISER (Tool for Eliciting Informative Structural Elements in RNA), a computational framework that captures both structural and sequence information contained in RNA sequences [25], to discover the structural RNA stability element (sRSE) regions bound by TARBP2.

### 2.2. Fluorescent In Situ Hybridization (FISH) Analysis

We purchased a tissue microarray with 28 GC tissue samples and paired adjacent normal tissues from Outdo Biotechnology (Shanghai, China).

We carried out FISH assays using a FISH Kit (RiboBio, Guangzhou, China) according to the manufacturer’s instructions. RiboBio Biotechnology (Guangzhou, China) designed and synthesized a unique probe targeting LINC01526. We captured and analyzed all microscopy images using a confocal laser microscope (Zeiss LSM710, Carl Zeiss, Oberkochen, Germany) [26,27].

### 2.3. RNA Extraction and Real-Time Quantitative PCR (RT-qPCR) Assays

We extracted total RNA from cells using the RNA isolator Total RNA Extraction Reagent (Vazyme, Nanjing, Jiangsu, China) according to the manufacturer’s instructions. Next, we reverse-transcribed RNA into cDNA using a HiScript III RT SuperMix qPCR kit (R323-01, Vazyme, Nanjing, China). We performed RT-qPCR using SYBR qPCR SuperMix Plus (Novoprotein Scientific Inc., Shanghai, China) on an Applied Biosystems 7500 Real-Time PCR system. We normalized the relative expression of genes to 18S ribosomal RNA (18srRNA) expression and analyzed it using the 2^−ΔΔCT^ method. The primers used in this study were:

- LINC01526-F, 5′-GGAAGGTCCTGCCCTTTGTT-3′; LINC01526-R, 5′-CTGTCCTATTCAGTGGGGGC-3′;
- TARBP2-F, 5′-GCCTGAGGACATTCCGGTTT-3′; TARBP2-R, 5′-TCACTGTGTACTCCGGCAAC-3′;
- GNG7-F, 5′-AAGCTCTCTGAACAACGGGG-3′; GNG7-R, 5′-CGCTCAATCCCGGCTTCTAT-3′;
- CDKN2A-F, 5′-CCAGAGGCAGTAACCATGCC-3′; CDKN2A-R, AAACTACGAAAGCGGGGTGG-3′;
- GSDMD-F, 5′-CATGGTTCTGGAAACCCCGT-3′; GSDMD-R, 5′-CCACACTCAGCGAGTACACA-3′
- POLG-F, 5′-AGGGGCATTGTTGCTTGTTG-3′; POLG-R, 5′-ACTGCCTTGGAGCAGGTTTAT-3′;
- 18srRNA-F, 5′-AAACGGCTACCACATCCAAG-3′; 18srRNA-R, 5′-CCTCCAATGGATCCTCGTTA-3′.

### 2.4. Cell Culture and Treatments

We purchased human GC cell lines (HGC-27, AGS, SNU-1, and Hs746T) and normal human gastric epithelial cells (GES-1) from the Institute of Biochemistry and Cell Biology of the Chinese Academy of Sciences (Shanghai, China). The cell lines were identified by STRs (Short Tandem Repeat) without mycoplasma contamination. We cultured all the cells using Roswell Park Memorial Institute (RPMI)-1640 medium (Gibco, Grand Island, NY, USA) containing 10% fetal bovine serum (ExCell Bio, New Zealand) and 1% penicillin-streptomycin (NCM Biotech, Suzhou, China) at 37 °C with 5% CO_2_ in a humidified incubator.

GenePharma (Shanghai, China) designed small interfering RNAs (siRNAs) targeting LINC01526, TARBP2, GNG7, and negative control and DNA plasmids. We transfected HGC-27 and AGS cells with the siRNAs and DNA plasmids using Lipofectamine 2000 (Invitrogen, Waltham, MA, USA) according to the manufacturer’s instructions. After 48 h post-transfection, we harvested cells for the further experiments, including transfection efficiency and function assay. The siRNA sequences were as follows:

- si-LINC01526 #1, 5′-GCUGGUUCUAGGACCAUAU-3′;
- si-LINC01526 #2, 5′-GCUCCUAAAGGUUCCUUUA-3′;
- si-TARBP2 #1, 5′-GCUGCCUAGUAUAGAGCAA-3′;
- si-TARBP2 #2, 5′-GCCCACCGCAAAGAAUUCA-3′;
- si-GNG7 #1, 5′-GAGCUACUGUGAGCAACAU-3′;
- si-GNG7 #2, 5′-CCACUAACAACAUAGCCCA-3′;
- si-NC, 5′-UUCUCCGAACGUGUCACGU-3′.

### 2.5. Cell Proliferation Assays

We assessed cell proliferation through Cell-Counting Kit-8 (CCK8), colony formation, and 5-ethynyl-2-deoxyuridine (EdU) assays in HGC-27 and AGS cells.

After being transfected with siRNAs for 48 h, cells were inoculated into 96-well plates (2500 cells/well). Next, we added the CCK8 solution (Beyotime Institute of Biotechnology, Nantong, China) to cells at 0, 24, 48, 72, and 96 h, and measured the absorbance at 450 nm with a microplate reader (Bio-Rad Model 680, Richmond, CA, USA) according to the standard procedure [24,28].

For the colony formation assay, we seeded the cells into six-well plates (800 cells/well) 48 h post-transfection. As the clones are visible at 2 weeks, we fixed the cells with methanol and stained them with 0.1% crystal violet (Beyotime Institute of Biotechnology, Nantong, China), as previously described [29,30].

For the EdU assay, we used a detection kit (Ribobio, Guangzhou, China) following the manufacturer’s instructions as previously described [29]. Briefly, we fixed the cells treated with 50 μM EdU labeling medium for 2 h with 4% paraformaldehyde and permeabilized them using 0.5% Triton X-100. Next, we added an anti-EdU working solution and a 4′,6-diamidino-2-phenylindole (DAPI) staining solution. Lastly, we observed EdU-positive cells under a confocal laser-scanning microscope (Zeiss LSM710, Carl Zeiss).

### 2.6. Apoptosis Assays

We assessed apoptosis by performing a terminal deoxynucleotidyl transferase-mediated dUTP nick-end-labeling (TUNEL) assay using a TUNEL Detection Kit (Vazyme, Nanjing, Jiangsu, China) as described previously [28,29]. We observed TUNEL-positive cells through a confocal laser-scanning microscope.

### 2.7. Transwell Assays

We evaluated the migration capacity of HGC-27 and AGS cells through Transwell assays. We seeded transfected cells with 300 μL of serum-free medium into the 24-well Transwell chamber with 8 μm pore size (Corning, Corning, NY, USA), and 700 μL of complete medium under the chamber. After 48 h, we fixed and stained the migrated cells under the chamber membrane surface.

### 2.8. Xenograft Mouse Model

We housed five-week-old BALB/c nude mice under specific pathogen-free conditions in individually ventilated cages with sterilized food and water. Each mouse received a subcutaneous injection of HGC-27 cells (5 × 10^6^ in 0.1 mL of phosphate-buffered saline (PBS)) stably transfected with sh-LINC01526 or an empty vector to the two armpits (*n* = 5). The sh-LINC01526 plasmid was constructed by GenePharma (Shanghai, China) with the sh-LINC01526 sequences cloned into the lentiviral vector 3 (LV3) vector. We routinely measured tumor size every three days. After 15 days, we sacrificed all the mice, collected the subcutaneous tumors, and fixed them in formalin for further experiments. We calculated the tumor volumes as follows: volume = 0.5 × length × width^2^. All procedures involving animals and their care were approved by the Animal Ethics Committee of Affiliated Hospital of Nanjing University of Chinese Medicine (No. 2020DW-35-02, approved on 14 December 2020).

To investigate cell metastasis formation in vivo, we established a tail vein injection model. We injected 3 × 10 ^6^ cells transfected with sh-LINC01526 or an empty vector in 0.1 mL of PBS into the tail vein of nude mice. Two months later, we euthanized the mice, collected lung tissues, and fixed them in formalin. Finally, we counted the lung nodules.

### 2.9. Hematoxylin and Eosin (HE) Staining and Immunofluorescence

To identify metastatic lesions among lung nodules, we performed HE staining. After dewaxing the paraffin sections, we stained nuclei with hematoxylin and the cytoplasm with eosin. Next, we sealed the sections with tree gum and observed the tissue structures under a microscope.

For immunofluorescence [30,31], we blocked the section undergoing antigen retrieval with 1 % bovine serum albumin and incubated it with primary antibodies and secondary antibodies successively. Finally, we captured images with a confocal laser-scanning microscope and analyzed them. We used the same primary antibodies as in our previous article [30].

### 2.10. RNA Pull-Down Assay

We transcribed LINC01526 with T7 RNA polymerase (Ambio Life, Shanghai, China) and labeled it with a Biotin RNA-Labeling Mix (Ambio Life, Shanghai, China) according to the manufacturer’s instructions. Next, we performed RNA pull-down using a Pierce Magnetic RNA-Protein Pull-Down Kit (Thermo Scientific, Waltham, MA, USA) as previously described [24]. Finally, we identified the proteins that interacted with LINC01526 by liquid chromatography–mass spectrometry (LC–MS/MS) analysis [32].

### 2.11. Western Blotting

We lysed the cells with a Radio Immunoprecipitation Assay solution (Beyotime Institute of Biotechnology, Nantong, China) containing 1% protease inhibitor phenyl methyl sulfonyl fluoride, separated the obtained proteins by 10% sodium dodecyl sulfate-polyacrylamide gel electrophoresis, and transferred them to polyvinylidene difluoride membranes (Millipore, Bedford, MA, USA). Then, we blocked the membranes with 5% skimmed milk for 1 h and incubated them with primary antibodies and then with horseradish peroxidase-conjugated secondary antibodies. Finally, we revealed the band signals via an enhanced chemiluminescent substrate and quantified them with Image-Pro plus. The anti-tubulin and anti-TARBP2 antibodies came from the Beyotime Institute of Biotechnology and Proteintech (Chicago, IL, USA).

### 2.12. RNA Immunoprecipitation (RIP) Assay

We performed the RIP assays using a Magna RIP RNA-binding Protein Immunoprecipitation Kit (Millipore) as previously described [24]. After treating the cells with the RIP lysis buffer, we incubated the extracted proteins with anti-TARBP2 (Proteintech) or anti-IgG (Abcam, Cambridge, MA, USA) antibodies. Finally, we analyzed the purified RNAs bound to beads by RT-qPCR.

### 2.13. RNA Sequencing

We extracted RNA from HGC-27 cells transfected with si-TARBP2 or si-NC (*n* = 3 in each group) and sequenced it with the 2 × 150 bp paired-end (PE150)-sequencing strategy on an Illumina NovaSeq™ 6000 platform following the vendor’s recommended protocol (San Diego, CA, USA). We selected differentially expressed genes (DEGs) based on a fold change > 2.0 and false discovery rate (FDR) < 0.05; then, we conducted Gene Ontology (GO) enrichment analyses.

### 2.14. RNA Stability Assays

We treated the cells transfected with siRNA or plasmid vectors with 1 μg/mL actinomycin D and collected them at different time points. We then evaluated the relative mRNA levels through RT-qPCR.

### 2.15. Statistical Analysis

We performed statistical comparisons using Student’s *t*-test (pairs of groups) or one-way analysis of variance (ANOVA; multiple groups) via GraphPad Prism 9.0 and presented the results as mean ± standard deviation (SD). We considered *p* values < 0.05 as indicating statistical significance. We repeated each experiment at least three times. Biological replicates are shown in figure legend for their different respective experiments.

## 3. Results

### 3.1. LINC01526 Is Significantly Upregulated in Human Gastric Cancer Tissues and Associated with a Poor Prognosis

**Figure 1:**
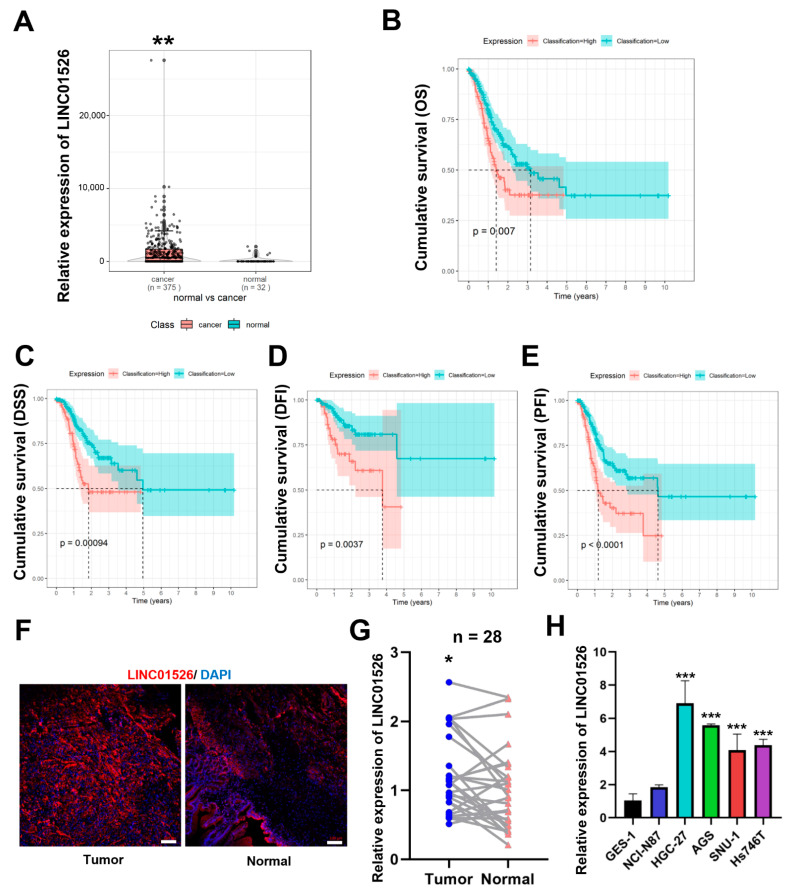
Relative LINC01526 expression in GC tissues and cell lines and its clinical significance. (**A**) Relative expression of LINC01526 in GC tissues (n = 375) and normal tissues (n = 32) based on TCGA datasets. (**B**–**E**) Kaplan–Meier curves for overall survival (OS), disease-free survival (DFS), disease-free interval (DFI), and progression-free interval (PFI) in GC patients. (**F**) The expression of LINC01526 (red) in gastric tissues and paired non-tumor tissues (n = 28) assessed by Fluorescence in Situ Hybridization (FISH) assay; nuclei were stained with DAPI (blue); scale bars: 100 μm. (**G**) Quantification of fluorescence intensity. (**H**) LINC01526 expression in human GC cell lines (NCI-N87, HGC-27, AGS, SNU-1, and Hs746T) and the normal human gastric epithelial cell line (GES-1) assessed by RT-qPCR; n = 3 in each group. * *p* < 0.05, ** *p* < 0.01, and *** *p* < 0.001.

Based on the RNA-sequencing dataset extracted from the TCGA database, we found that GC tissues (*n* = 375) expressed higher LINC01526 levels than normal tissues (*n* = 32) (Figure 1A). Furthermore, as the overall survival (OS), disease-free survival (DFS), disease-free interval (DFI), and progression-free interval (PFI) curves showed, a high LINC01526 expression was associated with shorter survival times in patients with GC (Figure 1B–E). Next, we performed a FISH assay on 28 paired human GC tissues and corresponding adjacent tissues and confirmed that LINC01526 was highly expressed in GC tissues and localized in the cytoplasm (Figure 1F,G). Furthermore, GC cell lines (HGC-27, AGS, SNU-1, and Hs746T) also expressed higher LINC01526 levels than normal human gastric epithelial cells (GES-1) (Figure 1H). According to these findings, LINC01526 may be essential to the regulation of GC carcinogenesis and progression.

### 3.2. Knockdown of LINC01526 Suppresses Gastric Cancer Cell Proliferation and Migration and Promotes Apoptosis In Vitro

**Figure 2:**
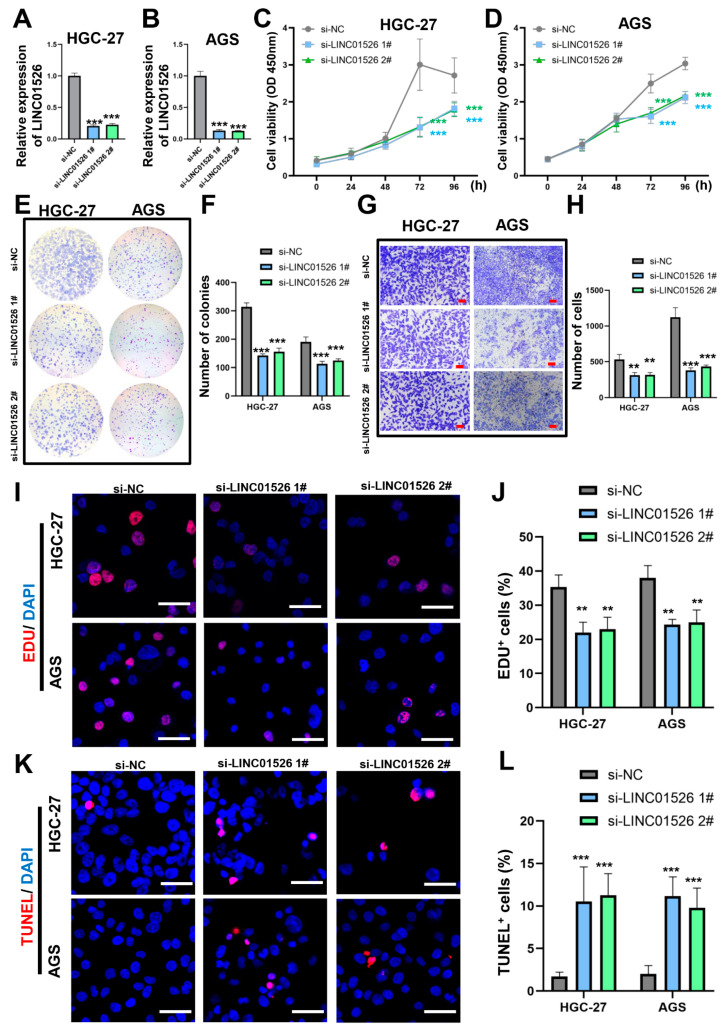
The effect of LINC01526 on GC cell proliferation, migration, and apoptosis in vitro. (**A**,**B**) Relative LINC01526 expression levels in (**A**) HGC-27 and (**B**) AGS cells transfected with si-NC or si-LINC01526 #1 or #2, assessed by RT-PCR; *n* = 3 in each group. (**C**,**D**) CCK8 assay (*n* = 6) results showing the viability of cells transfected with si-LINC01526. (**E**) Colony formation ability in cells transfected with si-LINC01526; *n* = 3 in each group. (**F**) Quantification of results from (**E**). (**G**) Transwell assays (*n* = 3) results showing cell migration ability; scale bars: 50 μm. (**H**) Quantification of results from (**G**). (**I**) EdU assay (*n* = 3) results showing cell proliferation; proliferating cells were labeled with EdU (red), and cell nuclei were stained with DAPI (blue); scale bars: 50 μm. (**J**) Quantification of results from (**I**). (**K**) TUNEL assay (*n* = 3) results showing apoptosis; apoptotic cells were labeled with TUNEL (red), and cell nuclei were stained with DAPI (blue); scale bars: 50 μm. (**L**) Quantification of results from (**K**). ** *p* < 0.01, and *** *p* < 0.001.

To document the biological features of LINC01526 in malignant GC progression in vitro, we performed loss-of-function experiments using HGC-27 and AGS cells, which express high LINC01526 levels (Figure 1H). The siRNAs si-LINC01526 #1 and #2 markedly and independently knocked down LINC01526 mRNA siRNAs (Figure 2A,B). Apparently, the viability of the cells transfected with si-LINC01526 diminished (Figure 2C,D). Additionally, the colony formation assays confirmed that knocking LINC01526 down inhibited GC cell colony formation (Figure 2E,F). Furthermore, the EdU, CCK8, and colony formation assays proved that downregulating LINC01526 impaired cell proliferation (Figure 2I,J). Next, the Transwell assays revealed that silencing LINC01526 suppressed the migratory capacity of HGC-27 and AGS cells (Figure 2G,H). In addition, the TUNEL assays confirmed that decreasing LINC01526 expression induced GC cell apoptosis (Figure 2K,L). These results suggest that knocking LINC01526 down suppresses GC cell proliferation and migration and promotes apoptosis in vitro.

### 3.3. Knockdown of LINC01526 Suppresses Gastric Cancer Cells Growth and Metastasis In Vivo

**Figure 3:**
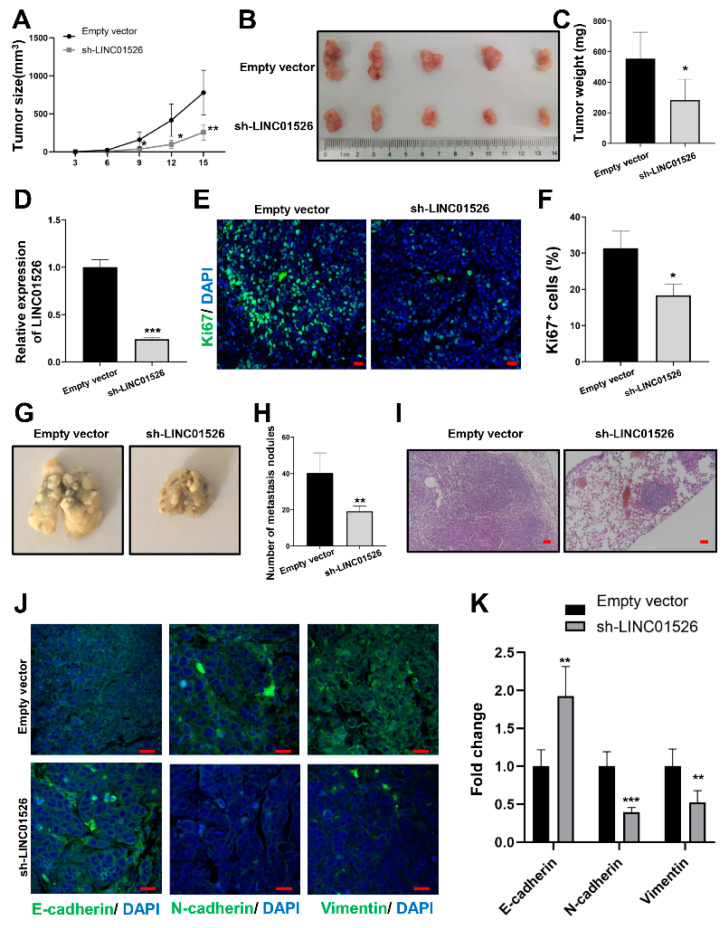
The effect of LINC01526 on GC cell proliferation and migration in vivo. (**A**) tumor volume, (**B**) macroscopic appearance, and (**C**) weight of tumors resulting from the subcutaneous injection of HGC-27 cells transfected with sh-LINC01526 or an empty vector into nude mice (n = 5). (**D**) Relative LINC01526 expression levels in tumors in the sh-LINC01526 and empty vector group, assessed by RT-PCR. (**E**) Ki-67 levels measured by immunofluorescence; scale bars: 20 μm. (**F**) Quantification of results from (**E**). (**G**) Lung tissues removed from nude mice 2 months after the injection of HGC-27 cells transfected with sh-LINC01526 or an empty vector into the tail vein (n = 5). (**H**) Lung nodule counts. (**I**) H&E staining of mouse lung tissues; scale bars: 100 μm. (**J**) Immunofluorescence staining of E-cadherin, N-cadherin, and vimentin; scale bars: 20 μm. (**K**) Quantification of (**J**). * *p* < 0.05, ** *p* < 0.01, and *** *p* < 0.001.

To further elucidate the role of LINC01526 in GC tumorigenesis in vivo, we established a xenograft tumor-bearing nude mouse model by injecting HGC-27 cells stably expressing sh-LINC01526 or an empty vector to nude mice (*n* = 5). During the xenograft tumor growth, we measured and calculated the tumor volumes, and found that the tumors grew much more slowly in the sh-LINC01526 group than in the empty vector group (Figure 3A). Fifteen days after the injection, we sacrificed the mice. The tumors derived from the sh-LINC01526 cells were much smaller and lighter than those from the empty vector group (Figure 3B,C). It was clearly seen that tumors from the sh-LINC01526 group expressed lower LINC01526 levels compared with the empty vector group (Figure 3D). Moreover, the immunofluorescence analysis revealed that the sh-LINC01526 group had fewer Ki67-positive cells than the empty vector group (Figure 3E,F), indicating that the LINC01526 knockdown suppressed GC cell growth in vivo.

Our next question was: how does LINC01526 affect GC metastasis in vivo? To answer this, we established a GC lung metastasis model by injecting HGC-27 cells transfected with sh-LINC01526 or an empty vector into the tail vein of nude mice (*n* = 5). Unsurprisingly, after 2 months, the sh-LINC01526 group had strikingly fewer lung metastases nodules than the empty vector group (Figure 3G,H). Similarly, the H&E staining suggested that the lung nodules derived from the tumors (Figure 3I). To evaluate whether the epithelial-mesenchymal transition played a part in the invasiveness of GC, we monitored specific epithelial–mesenchymal transition markers such as E-cadherin, N-cadherin, and vimentin. We observed more E-cadherin-positive cells and fewer N-cadherin- and vimentin-positive cells in the sh-LINC01526 group than in the empty vector group (Figure 3J,K).

Taken together, these results suggest that knocking LINC01526 down suppresses GC cell growth and metastasis in vivo.

### 3.4. LINC01526 Interacts with TARBP2, and TARBP2 Functions as an Oncogene in GC Cells

**Figure 4:**
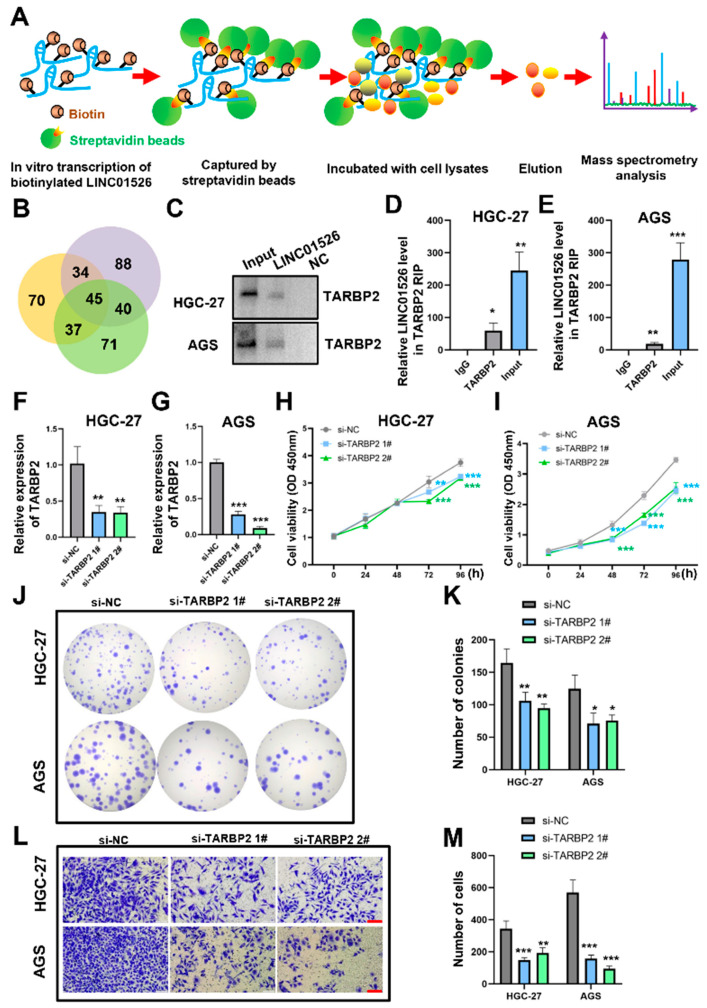
LINC01526 interacts with TARBP2, and TARBP2 acts as an oncogene in GC. (**A**) Flow chart of the RNA pull-down assays. (**B**) Venn diagram of proteins identified by mass spectrometry from three independent RNA pull-down assays. (**C**) Western blot showing the TARBP2 levels after the LINC04526 pull-down assay (n = 3 for each group). The uncropped blots are shown in Figure S2. (**D**,**E**) Relative LINC01526 levels in TARBP2 RNA immunoprecipitation (RIP) assays in HGC-27 and AGS cells (quantified by RT-qPCR). Rabbit IgG was the negative control for immunoprecipitation (n = 3 in each group). (**F**,**G**) Relative TARBP2 expression levels in HGC-27 and AGS cells transfected with si-NC or si-TARBP2 #1 or #2 quantified by RT-qPCR; n = 3 in each group. (**H**,**I**) CCK8 assay (n = 6) results showing the viability of cells transfected with si-TARBP2. (**J**) Colony formation ability of cells transfected with si-TARBP2; n = 3 in each group. (**K**) Quantification of results from (**J**). (**L**) Transwell assay (n = 3) results showing cell migration ability; scale bars: 100 μm. (**M**) Quantification of (**L**). * *p* < 0.05, ** *p* < 0.01, and *** *p* < 0.001.

To further explore the potential molecular mechanism of LINC01526 in GC, we performed an RNA pull-down-LC-MS/MS assay and identified the potential proteins interacting with LINC01526 in the GC cells (Figure 4A). The Venn diagram analysis revealed 45 overlapping proteins from three independent RNA pull-down assays (Figure 4B). The most meaningful of these proteins was TARBP2, which had more unique peptides than the other identified proteins (Table S1). Furthermore, Western blotting and RIP analysis confirmed this abundance of binding sites in the HGC-27 and AGS cells (Figure 4C–E).

In addition, the anti-TARBP2 siRNAs downregulated TARBP2 in the HGC-27 and AGS cells (Figure 4F,G). Unsurprisingly, both the CCK8 and colony formation assays confirmed that downregulating TARBP2 reduced GC cell proliferation (Figure 4H–K). The Transwell assays confirmed that silencing TARBP2 suppressed the migratory capacity of both the HGC-27 and AGS cells (Figure 4L,M). Finally, we analyzed TARBP2 expression in GC through GEPIA database (http://gepia2.cancer-pku.cn/, accessed on 23 September 2022). The result showed that TARBP2 was overexpressed in GC tissue compared with normal tissue (Figure S1A).

### 3.5. GNG7 Is the Potential Downstream Target mRNA of TARBP2 in GC Cells

**Figure 5:**
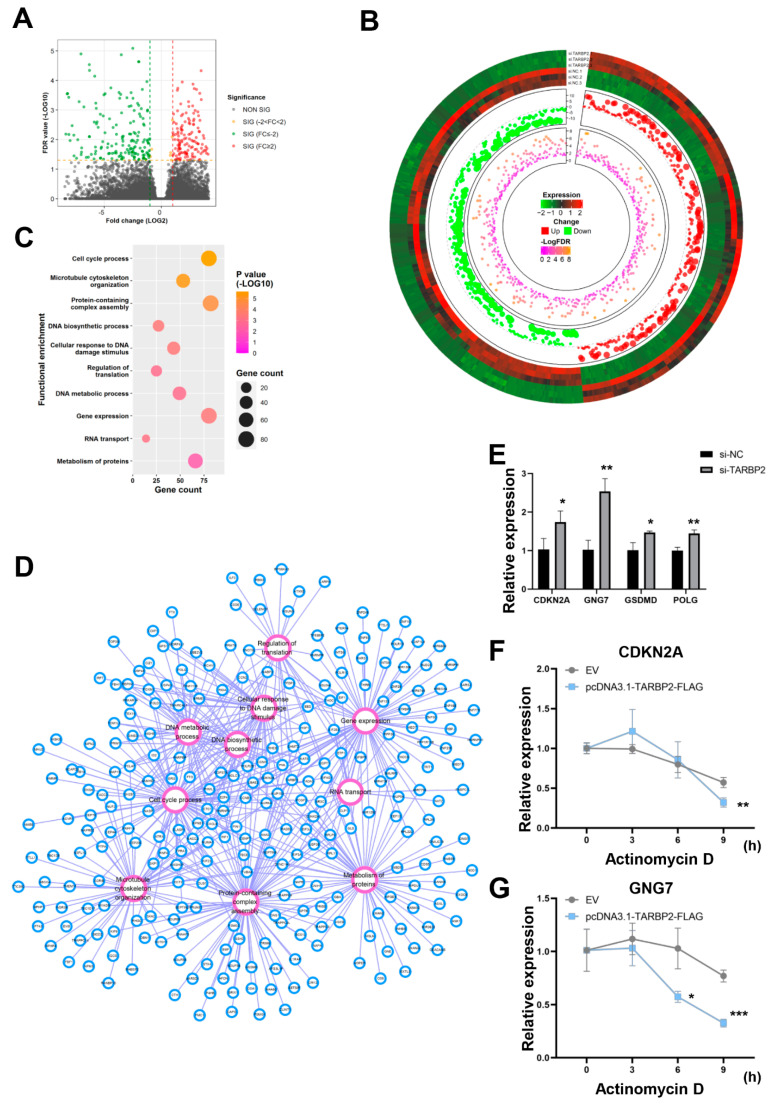
The potential downstream target mRNA of TARBP2 in GC cells. (**A**,**B**) Volcano plot and circular heatmap showing the differentially expressed transcripts between the si-TARBP2- and si-NC-transfected HGC-27 cells based on RNA-sequencing data (fold change > 2.0 and FDR < 0.05). (**C**) The KEGG pathways and GO biological processes enriched in the differentially expressed genes. (**D**) Network diagram of functional enrichment: purple circles represent function, blue circles represent gene, and lines represent relationships between gene and function. (**E**) The mRNA expression levels of candidate tumor suppressors (based on the enriched pathways) in si-TARBP2-transfected HGC-27 cells (n = 3). (**F**,**G**) RNA stability assay results showing the degradation rates of CDKN2A and GNG7 in HGC-27 cells transfected with pcDNA3.1-TARBP2-FLAG or an empty vector (EV) (n = 3). * *p* < 0.05, ** *p* < 0.01, and *** *p* < 0.001.

TARBP2, a double-stranded, RNA-binding protein [33], binds and destabilizes its target mRNAs [34]. We identified the tumor suppressors repressed by TARBP2 through RNA sequencing. After a TARBP2 knockdown in the HGC-27 cells, we found 313 upregulated transcripts and 349 downregulated transcripts (Figure 5A,B, and Table S2). Afterward, the GO analysis revealed that these DEGs were enriched in many biological processes, including the “cell cycle process”, “microtubule cytoskeleton organization”, “protein-containing complex assembly”, “DNA biosynthetic process”, “cellular response to DNA damage stimulus”, “regulation of translation”, “DNA metabolic process”, “gene expression”, “RNA transport”, and “metabolism of proteins” (Figure 5C,D). Among these DEGs, CDKN2A [35,36,37], GNG7 [38], GSDMD [39,40,41,42], and POLG [43] are known tumor suppressors in GC. An RT-qPCR analysis confirmed that GNG7 and CDKN2D were sharply overexpressed after the TARBP2 knockdown in the HGC-27 cells (Figure 5E). Subsequently, to confirm the target gene of TARBP2, we carried out RNA stability assays. In the HGC-27 cells treated with actinomycin D, upregulating TARBP2 dramatically decreased the GNG7 mRNA’s half-life, and had a minor effect on the CDKN2A mRNA’s stability (Figure 5F,G). Moreover, based on the GEPIA database, we found that the expression of GNG7 in GC was lower than normal tissues (Figure S1B). Thus, we speculated that GNG7 is the major downstream target mRNA of TARBP2 in GC cells.

### 3.6. “LINC01526-TARBP2” Decreased GNG7 mRNAs Stability in GC Cells

**Figure 6:**
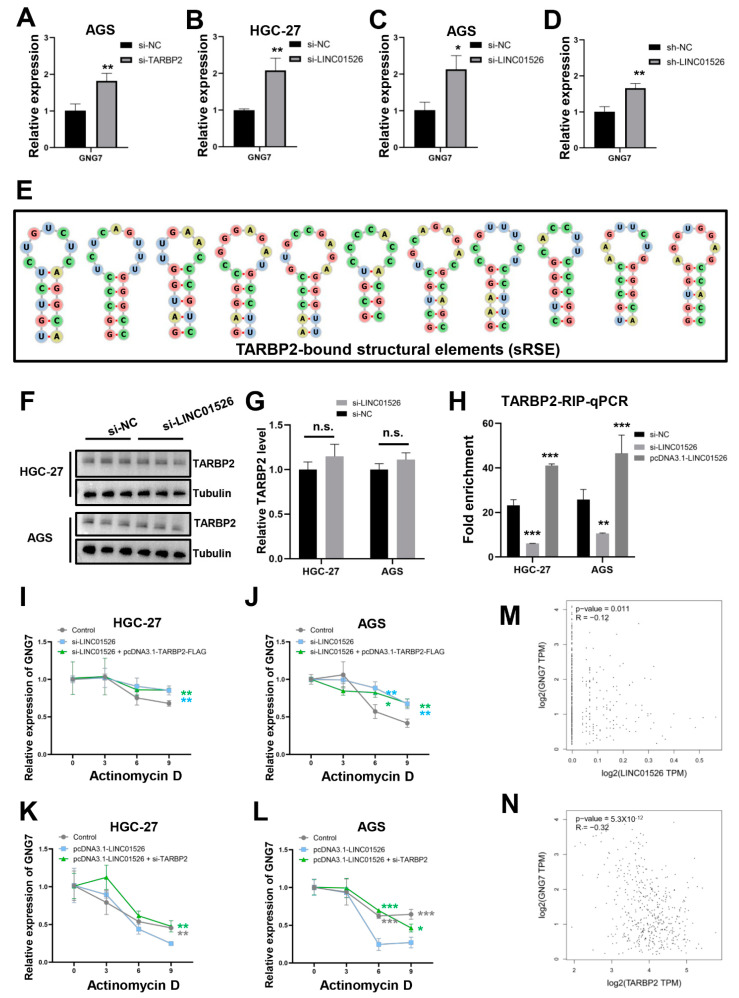
The effect of “Linc01526-TARBP2” on GNG7. (**A**) The mRNA expression levels of GNG7 in AGS cells transfected with si-TARBP2 or si-NC (*n* = 3). (**B**,**C**) The mRNA expression levels of GNG7 in HGC-27 and AGS cells transfected with si-LINC01526 or si-NC (*n* = 3). (**D**) The mRNA expression levels of GNG7 in subcutaneous tumors from the empty vector and the sh-LINC01526 group (n = 3). (**E**) The predicted TARBP2-binding structural elements (sRSE) in the 3′UTR region of GNG7 mRNA. (**F**,**G**) Western blot (n = 3) showing the expression of TARBP2 in HGC-27 and AGS cells transfected with si-LINC01526 or si-NC. The uncropped blots are shown in Figure S3. (**G**) Quantification of results from (**F**). (**H**) RIP-qPCR confirmed that TARBP2 binds GNG7 mRNA in HGC-27 and AGS cells and that LINC01526 affects this interaction. (**I**,**J**) RNA stability assay results showing the degradation rates of GNG7 in HGC-27 and AGS cells transfected with si-LINC01526, si-LINC01526, and pcDNA3.1-TARBP2-FLAG, or si-NC + EV (n = 3). (**K**,**L**) RNA stability assay results showing the degradation rates of GNG7 in HGC-27 and AGS cells transfected with pcDNA3.1-LINC01526, pcDNA3.1-LINC01526 combined with si-TARBP2, or EV + si-NC (*n* = 3). (**M**,**N**) Analysis of the relationship between GNG7 and (**M**) LINC01526 or (**N**) TARBP2 expression. * *p* < 0.05, ** *p* < 0.01, and *** *p* < 0.001.

Downregulating TARBP2 and LINC01526 in HGC-27 and AGS cells, respectively, significantly upregulated GNG7 (Figure 5E and Figure 6A–C). Consistently, the tumors derived from the sh-LINC01526 group also expressed higher levels of GNG7 (Figure 6D). However, what kind of functional connections do they have?

According to previous studies, TARBP2 preferentially binds double-stranded RNAs (e.g., stem-loops), with high GC closely resembling sRSE (GC-rich structural cis-regulatory RNA elements) [44]. Goodarzi et al. confirmed that TARBP2 negatively regulates the stability of amyloid precursor protein (APP) and zinc finger protein 395 (ZNF395) by binding sRSEs in the 3′UTR (untranslated regions) and promotes cancer [34]. To identify potential TARBP2-binding RNAs in GC, we employed TEISER, a framework for identifying the structural motifs that are informative of whole-genome measurements across all the transcripts [25]. Consistent with our estimate, we found eleven sRSEs in the 3′UTR region of GNG7 that potentially binds to TARBP2 (Figure 6E and Table S3).

More importantly, the protein level of TARBP2 remained virtually unchanged (Figure 6F,G), but the RIP assay confirmed that markedly reduced amounts of GNG7 were precipitated with TARBP2 in the LINC01526-knockdown HGC-27 and AGS cells (Figure 6H). Conversely, an LINC01526 overexpression increased the amount of GNG7 bound to TARBP2 in the HGC-27 and AGS cells (Figure 6H). Subsequently, the RNA stability assays confirmed that downregulating LINC01526 increased the GNG7 mRNA half-life and overexpressing TARBP2 did not attenuate this increase in the HGC-27 and AGS cells (Figure 6I,J), suggesting that LINC01526 is required for TARBP2-mediated GNG7 mRNA decay. Furthermore, overexpressing LINC01526 decreased the GNG7 mRNA half-life and si-TARBP2 could rescue this change (Figure 6K,L). This finding demonstrated that LINC01526 affected the GNG7 mRNA stability in a manner dependent on TARBP2. Finally, the expression levels of GNG7 were negatively correlated with both LINC01526 and TARBP2 expression levels in the GC tissues (Figure 6M,N).

### 3.7. LINC01526 Interacts with TARBP2, thereby Regulating GNG7 and Promoting GC Cells’ Proliferation and Migration

**Figure 7:**
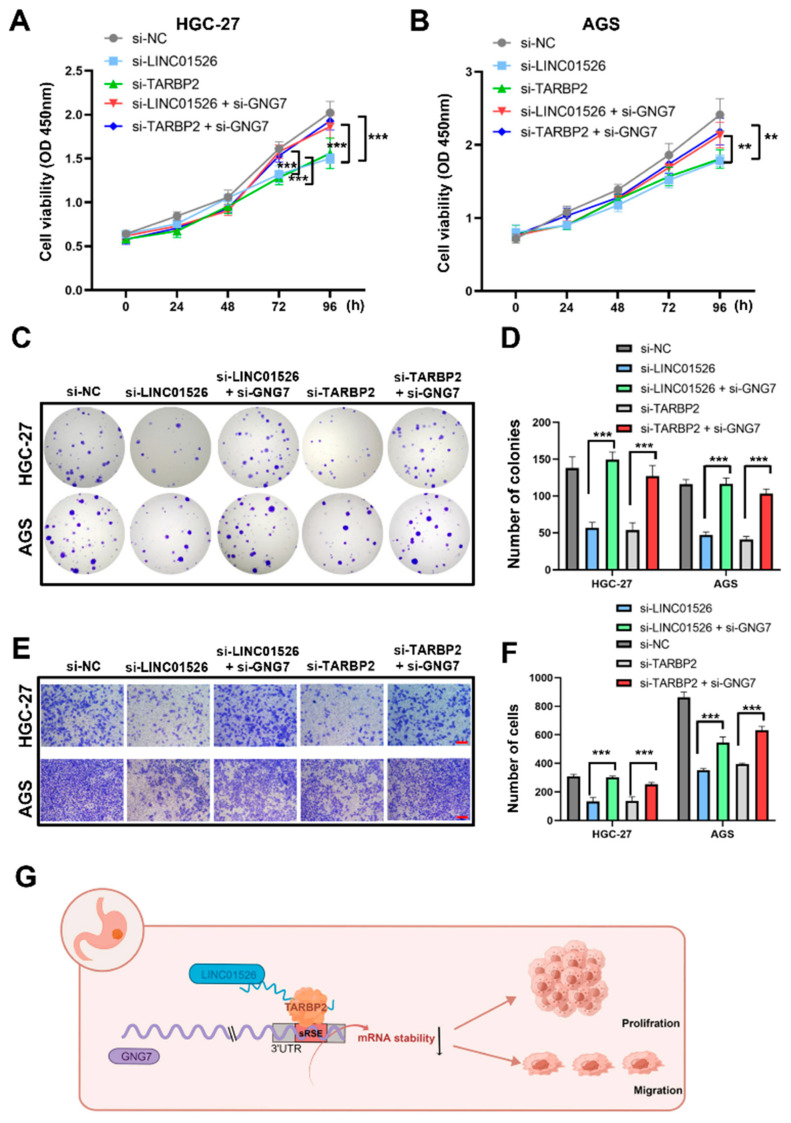
Inhibiting GNG7 reverses the regulatory effect of LINC01526 and TARBP2 in GC cells. (**A**,**B**) CCK8 assay results showing cell viability in HGC-27 and AGS cells transfected with si-LINC01526 or si-TARBP2, alone or co-transfected with si-GNG7 (n = 6). (**C**) Colony formation assay results showing cell proliferation (n = 3). (**D**) Quantification of results from (**C**). (**E**) Transwell assay results showing cell migration (n = 3), scale bars: 100 μm. (**F**) Quantification of results from (**E**). (**G**) Schematic diagram of the mechanism by which LINC01526 promotes tumor proliferation and migration in GC cells. ** *p* < 0.01, and *** *p* < 0.001.

To further explore the biological relationship between LINC01526/TARBP2 and GNG7 in GC progression, we carried out a rescue experiment. We transfected the HGC-27 and AGS cells with si-LINC01526 or si-TARBP2, both alone or co-transfected with si-GNG7. The CCK8 and colony formation assays revealed that downregulating GNG7 partially rescued the cell proliferation inhibited by LINC01526 or TARBP2’s silencing (Figure 7A–D). Additionally, the Transwell assay indicated that co-transfection significantly increased the migratory cell count (Figure 7E,F).

Taken together, our results demonstrate that LINC01526 interacts with TARBP2, thereby regulating GNG7 mRNA stability and expression, and eventually promoting GC cell proliferation and migration (Figure 7G).

## 4. Discussion

The difficulty of early diagnosis and the development of drug resistance contribute to the high mortality rate associated with GC [45]. Several lncRNAs can promote or inhibit the initiation and migratory progression of GC in various ways [8,9]. Many lncRNAs have been identified as potential biomarkers for a GC diagnosis and therapy [46,47]. Among those, bioinformatics analyses have identified LINC01526 and 12 other lncRNAs as prognostic markers for GC [23], but the functions and mechanisms of LINC01526 in GC remain elusive. The present study confirmed, through bioinformatic, RT-qPCR, and FISH analyses, that LINC01526 was significantly upregulated in human GC tissues and associated with a poor outcome. Moreover, the loss-of-function assay confirmed that knocking down LINC01526 suppressed GC cell proliferation and migration in vitro and in vivo, suggesting that LINC01526 promotes GC progression.

LncRNAs regulate gene expression through gene modifications, histone modifications, and chromatin remodeling without altering the DNA sequence at the pre-transcriptional level [48,49]. Moreover, they act as competing endogenous RNAs, sequestering micro RNAs to regulate their translation [50], or interact with specific proteins to maintain mRNA stability or induce mRNA decay at the post-transcriptional level [51,52,53,54]. In this article, the RNA pull-down and RIP assays demonstrated the physical connection between LINC01526 and TARBP2.

TARBP2, a double-stranded, RNA-binding protein, interacts with GC-rich stem-loops in the 3′UTR region [55] and is a functional component of the RNA interference silencing complex. Previous studies have proved that TARBP2 participates in viral infections [33], micro RNA biogenesis [56], and tumorigenesis [57]. TARBP2 might act as a tumor promoter in lung cancer [34], breast cancer [58], melanoma [59], hepatocellular carcinoma [60], adrenocortical carcinoma [61], and diffuse large B-cell lymphoma [62], but as a tumor suppressor in osteosarcoma [63] and Ewing sarcoma [64]. However, the role and mechanism of TARBP2 in GC progression had remained uncertain. The CCK8, colony formation, and Transwell assays jointly confirmed that TARBP2 promotes GC cell proliferation and migration in vitro.

Our RNA sequencing, GO analysis, RNA stability assays, and TEISER analysis demonstrated that TARBP2 targets GNG7 mRNA. GNG7, a member of the large G protein gamma family, is related to transmembrane-signaling pathways and is engaged in cell contact-induced growth arrest, thereby suppressing uncontrolled cell proliferation [65,66]. A large body of evidence indicates that GNG7 is downregulated in various tumors, including esophageal cancer [67], lung adenocarcinoma [68], clear cell renal cell carcinoma [69], etc. In gastrointestinal tract cancer, GNG7 suppresses cell growth in vitro and in vivo by upregulating the expression of the cyclin-dependent kinase inhibitor p27Kip1 [66]. In our study, LINC01526 only enhanced the binding of TARBP2 to GNG7 but did not affect TARBP2 protein levels. Furthermore, silencing LINC01526 increased GNG7 mRNA stability. Vitally, the capacity of TARBP2 to bind and decay GNG7 mRNA depended on LINC01526, and LINC01526 decays GNG7 mRNA through targeting TARBP2. In addition, the expression levels of GNG7 were negatively correlated with both LINC01526 and TARBP2 in the GC tissues. Finally, the rescue experiment confirmed the role of the LINC0526/TARBP2/GNG7 axis in GC.

In this study, some limitations exist in the knockdown experiments in vitro using siRNA, which yields a transient knockdown and is also associated with multiple off-target effects. Both stable knockdown experiments using lentiviral vectors and knockout experiments in the future will be more credible. Additionally, they will be employed in a further study.

## 5. Conclusions

Overall, this study has shown that LINC01526 was upregulated in GC tissues. The elevated LINC01526 levels promoted GC cell proliferation and migration in vitro and in vivo and were associated with poor outcomes in patients. Furthermore, LINC01526 interacted with TARBP2, and “LINC01526-TARBP2” bound and decayed GNG7 mRNA. Finally, knocking down GNG7 rescued the cell proliferation and migration inhibited by LINC01526 or TARBP2’s silencing.

In conclusion, LINC01526 promotes GC progression through interacting with TARBP2, thereby inducing GNG7 mRNA degradation. The mechanistic characterization of LINC01526 may help to shed light on the future development of lncRNA-based GC therapies.

## Data Availability

The processed data that support the findings of this study are available from the corresponding author.

## Supplementary Materials

The following supporting information can be downloaded at: https://www.mdpi.com/article/10.3390/cancers14194940/s1, Figure S1. Relative TARBP2 and GNG7 expression in GC tissue; Figure S2. Uncropped Western Blot images for Figure 4C; Figure S3. Uncropped Western Blot images for Figure 6; Table S1. Putative interactors identified from three independent RNA pull-down assays in this study, Table S2. Differentially expressed genes identified in this study, Table S3. sRSEs in the 3′UTR of GNG7.
