# Supplementary material for: LINC01526 Promotes Proliferation and Metastasis of Gastric Cancer by Interacting with TARBP2 to Induce GNG7 mRNA Decay
Source: Cancers (Basel). 2022 Oct 9;14(19):4940. doi: 10.3390/cancers14194940 (PMC9562272; doi:10.3390/cancers14194940)
Supplement: Supplementary file 1 [file cancers-14-04940-s001.zip › Table S3.pdf]

Supplementary Table S3. sRSEs in the 3 'UTR of GNG7

Index	Sequence	Length	3UTR Start	3UTR End	Stem loop	GC ratio
1	UGUCUCUGUCUCAGGCA	17	59	75	(((((.......)))))	52.94%
2	GGCCCUCUCAGUUUGGGCC	19	238	256	(((((.........)))))	68.42%
3	GAUGGUUGAACCCGUC	16	583	598	(((((......)))))	56.25%
4	GAGGCCGGGAGAUGCCUU	18	1424	1441	(((((........)))))	66.67%
5	AACCCGUGCCGAGAGGGUU	19	1744	1762	(((((.........)))))	63.16%
6	GCGUCCCCACCACGC	15	2116	2130	((((.......))))	80.00%
7	GCUGCUGCAGAGACGCAGC	19	2401	2419	(((((.........)))))	68.42%
8	GAAGGCCGUUUCUCCUUC	18	2967	2984	(((((........)))))	55.56%
9	CUGGGCACCUUCCCGG	16	3087	3102	(((((......)))))	75.00%
10	UGCCCAAGUUCUGGGGCA	18	3376	3393	(((((........)))))	61.11%
11	GGUGGAGGUGGAGCCACC	18	3451	3468	(((((........)))))	72.22%
