# Supplementary figures and images for: LINC01526 Promotes Proliferation and Metastasis of Gastric Cancer by Interacting with TARBP2 to Induce GNG7 mRNA Decay
Source: Cancers (Basel). 2022 Oct 9;14(19):4940. doi: 10.3390/cancers14194940 (PMC9562272; doi:10.3390/cancers14194940)

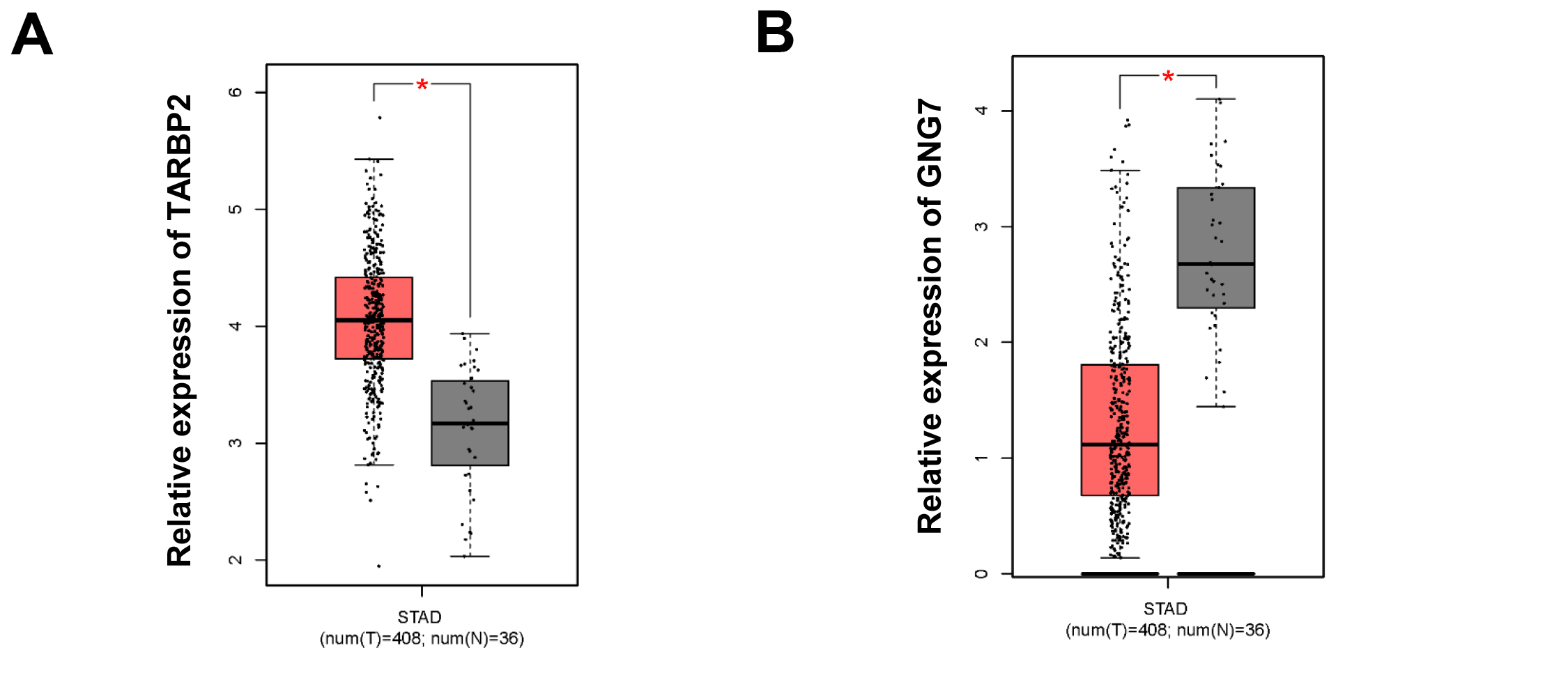

Supplement: Supplementary file 1 [file cancers-14-04940-s001.zip › Figure S1.tif]

**Fig 4C, TARBP2, HGC-27**

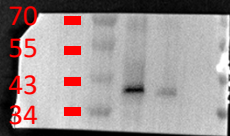

**Fig 4C, TARBP2, AGS**

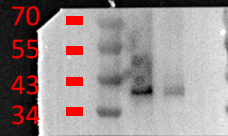

Supplement: Supplementary file 1 [file cancers-14-04940-s001.zip › Figure S2.pdf]

**Fig 6E, Tubulin, HGC-27**

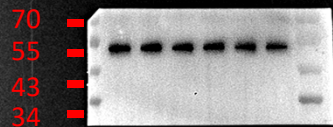

**Fig 6E, TARBP2, HGC-27**

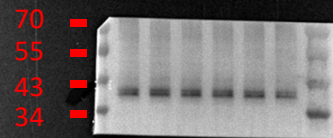

**Fig 6E, Tubulin, AGS**

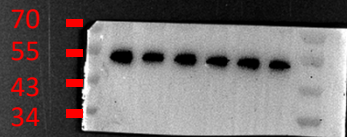

**Fig 6E, TARBP2, AGS**

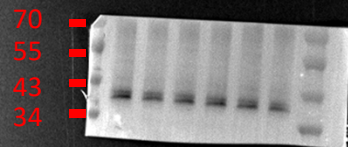

Supplement: Supplementary file 1 [file cancers-14-04940-s001.zip › Figure S3.pdf]
